# Intact spermatogenesis in an azoospermic patient with AZFa (sY84 and sY86) microdeletion and a homozygous TG12-5T variant in *CFTR*

**DOI:** 10.1186/s12610-025-00260-7

**Published:** 2025-04-01

**Authors:** Yifan Sun, Beifen Zhong, Zizhou Meng, Yuxiang Zhang, Zheng Li, Chencheng Yao

**Affiliations:** 1 Department of Andrology, Shanghai Key Laboratory of Reproductive Medicine, The Center for Men’s Health, Urologic Medical Center, Shanghai General Hospital, Shanghai Jiao Tong University School of Medicine, Shanghai, 200080 China; 2 State Key Laboratory of Reproductive Medicine and Offspring Health, School of Clinical Medicine, The Affiliated Taizhou People’s Hospital of Nanjing Medical University, Nanjing Medical University, Taizhou, 225300 China

**Keywords:** Azoospermia, AZFa microdeletion, Cystic fibrosis transmembrane conductance regulator gene, Spermatogenesis, Congenital bilateral absence of the vas deferens, Azoospermie, Microdélétion AZFa, Mucoviscidose, Gène régulateur de la Conductance transmembranaire, Spermatogenèse, Absence bilatérale congénitale du Canal Déférent

## Abstract

**Background:**

Azoospermia, the most severe form of male infertility, is categorized into two types: non-obstructive azoospermia (NOA) and obstructive azoospermia (OA), which exhibit significant genetic heterogeneity. Azoospermia factor (AZF) deletion is a common cause of NOA, whereas congenital bilateral absence of the vas deferens (CBAVD), a severe subtype of OA, is frequently linked to cystic fibrosis transmembrane conductance regulator (CFTR) gene variants. This case report is the first to document the coexistence of a partial AZFa microdeletion and a homozygous CFTR variant in a CBAVD-affected azoospermic patient with intact spermatogenesis.

**Case presentation:**

A 32-year-old man presented with primary infertility and azoospermia. Clinical evaluation revealed CBAVD (normal hormone levels, low semen volume, pH 6.0, and absence of the vas deferens). Genetic analysis accidentally revealed a 384.9 kb AZFa deletion (sY84 and sY86, but not sY1064, 1182) that removed USP9Y but retained DDX3Y in the proband, his fertile brother, and his father. A homozygous CFTR variant (TG12-5T) was also detected in the proband and his brother and was inherited from heterozygous parental carriers. Microdissection testicular sperm extraction (micro-TESE) revealed intact spermatogenesis, confirmed by histology and immunofluorescence, indicating normal germ cell development.

**Conclusion:**

This case expands the intricate genetic spectrum of azoospermia by illustrating the critical role of DDX3Y in the AZFa region in spermatogenesis and the variable penetrance of CFTR variant (TG12-5T) in CBAVD. These insights may refine diagnostic strategies and underscore the necessity for tailored fertility management in individuals with multifactorial genetic anomalies.

**Supplementary Information:**

The online version contains supplementary material available at 10.1186/s12610-025-00260-7.

## Résumé

**Contexte:**

L’azoospermie, la forme la plus grave d’infertilité masculine, est classée en deux types : l’azoospermie non obstructive (NOA) et l’azoospermie obstructive (OA), qui présentent une hétérogénéité génétique importante. La délétion du facteur d’azoospermie (AZF) est une cause fréquente de NOA, tandis que l’absence bilatérale congénitale du canal déférent (CBAVD), un sous-type sévère d’OA, est fréquemment liée à des variants du gène du régulateur de la conductance transmembranaire de la mucoviscidose (CFTR). Cette étude d’un Cas Clinique est la première à documenter la coexistence d’une microdélétion partielle d’AZFa et d’une variante homozygote de CFTR chez un patient azoospermique porteur d’une CBAVD avec une spermatogenèse intacte.

**Présentation du Cas:**

Un homme de 32 ans s’est présenté avec une infertilité primaire et une azoospermie. L’évaluation clinique a révélé une CBAVD (taux d’hormones normaux, faible volume de sperme, pH 6,0 et absence des canaux déférents). L’analyse génétique a accidentellement révélé une délétion d’AZFa de 384,9 kb (sY84 et sY86, mais pas sY1064, 1182) qui a éliminé USP9Y, mais a conservé DDX3Y chez le patient, son frère fertile et son père. Un variant homozygote de CFTR (TG12-5T) a également été détecté chez le patient et chez son frère, qui a été hérité de porteurs parentaux hétérozygotes. L’extraction testiculaire de spermatozoïdes par microdissection (micro-TESE) a révélé une spermatogenèse intacte, confirmée par histologie et immunofluorescence, indiquant un développement normal des cellules germinales.

**Conclusion:**

Ce cas élargit le spectre génétique complexe de l’azoospermie en illustrant le rôle critique dans la spermatogenèse de DDX3Y dans la région AZFa, et la pénétrance variable du variant CFTR (TG12-5T) dans la CBAVD. Ces informations peuvent affiner les stratégies de diagnostic et souligner la nécessité d’une gestion de la fertilité adaptée chez les personnes atteintes d’anomalies génétiques multifactorielles.

## Introduction

Male infertility is a multifactorial pathological condition [1]. The most severe form of male infertility is azoospermia, which is highly heterogeneous and has a broad genetic basis [2]. Azoospermia can be classified into two types: non-obstructive azoospermia (NOA) and obstructive azoospermia (OA).

Azoospermia factor (AZF) deletion of the Y chromosome is the second most common genetic cause of male infertility, following Klinefelter syndrome, which affects 5–10% % of azoospermic patients [3, 4]. The AZF region is divided into three subregions (AZFa, AZFb, and AZFc) on the basis of various phenotypes [5]. Among them, the AZFa region, which spans 792 kb, contains two genes associated with spermatogenesis: *USP9Y* and *DDX3Y*. Complete AZFa deletion, which accounts for 1% of AZF deletions, results from nonallelic homologous recombination (NAHR) between two HERV sequences [6–8]. This deletion leads to Sertoli cell-only (SCO) syndrome, which is characterized by the absence of germ cells in the seminiferous tubules, indicating a complete lack of sperm production [9]. Basic marker analysis of the AZFa region typically involves detecting two sequence-tagged sites (STSs), sY84 and sY86, positioned upstream of the *USP9Y* and *DDX3Y* genes. When both sY84 and sY86 are deleted, the probability of complete AZFa deletion is quite high; however, three cases of partial AZFa deletions encompassing sY84 and sY86 have been identified in oligozoospermic or even normozoospermic men [10–12]. These partial AZFa deletions did not result from NAHR and can be inherited by offspring.

Congenital bilateral absence of the vas deferens (CBAVD), a severe type of OA, is frequently associated with variants in genes such as *CFTR*, *ADGRG2*, and *SLC9A3* [13–16]. *CFTR*, the most classical variant, is located on the long arm of chromosome 7 (7q31) and comprises 27 exons. It encodes a chloride channel critical for electrolyte and fluid balance in various tissues, including the male reproductive system. *CFTR* variants are classified into six classes (I-VI) on the basis of defects in protein synthesis, protein processing, quantity, and stability [17]. These variants can manifest as a spectrum of conditions, from asymptomatic to cystic fibrosis (CF) and CFTR-related disorders (CFTR-RDs), such as CBAVD. Notably, the poly T tract in *CFTR* intron 9 (formerly known as intron 8), with 5 T, 7 T, and 9 T variants, significantly influences the quantity of functional CFTR protein [18]. While 7 T and 9 T variants are generally benign, the 5 T variant exhibits variable penetrance and can lead to reduced CFTR quantity. The 5 T variant is also modulated by the TG tract, a short string of TG repeats located immediately 5' to the poly T tract (typically 11, 12, or 13 repeats).

Herein, we present a unique case of a CBAVD-affected patient with intact spermatogenesis harboring a partial AZFa deletion (*USP9Y* deletion, with *DDX3Y* present) in combination with a homozygous *CFTR* variant (TG12-5 T). Our study revealed that *DDX3Y,* rather than *USP9Y,* is essential for spermatogenesis. Additionally, our study has broadened the variant spectrum of azoospermia.

## Case presentation

### Clinical and diagnostic evaluation revealed cbavd and OA

**Fig. 1:**
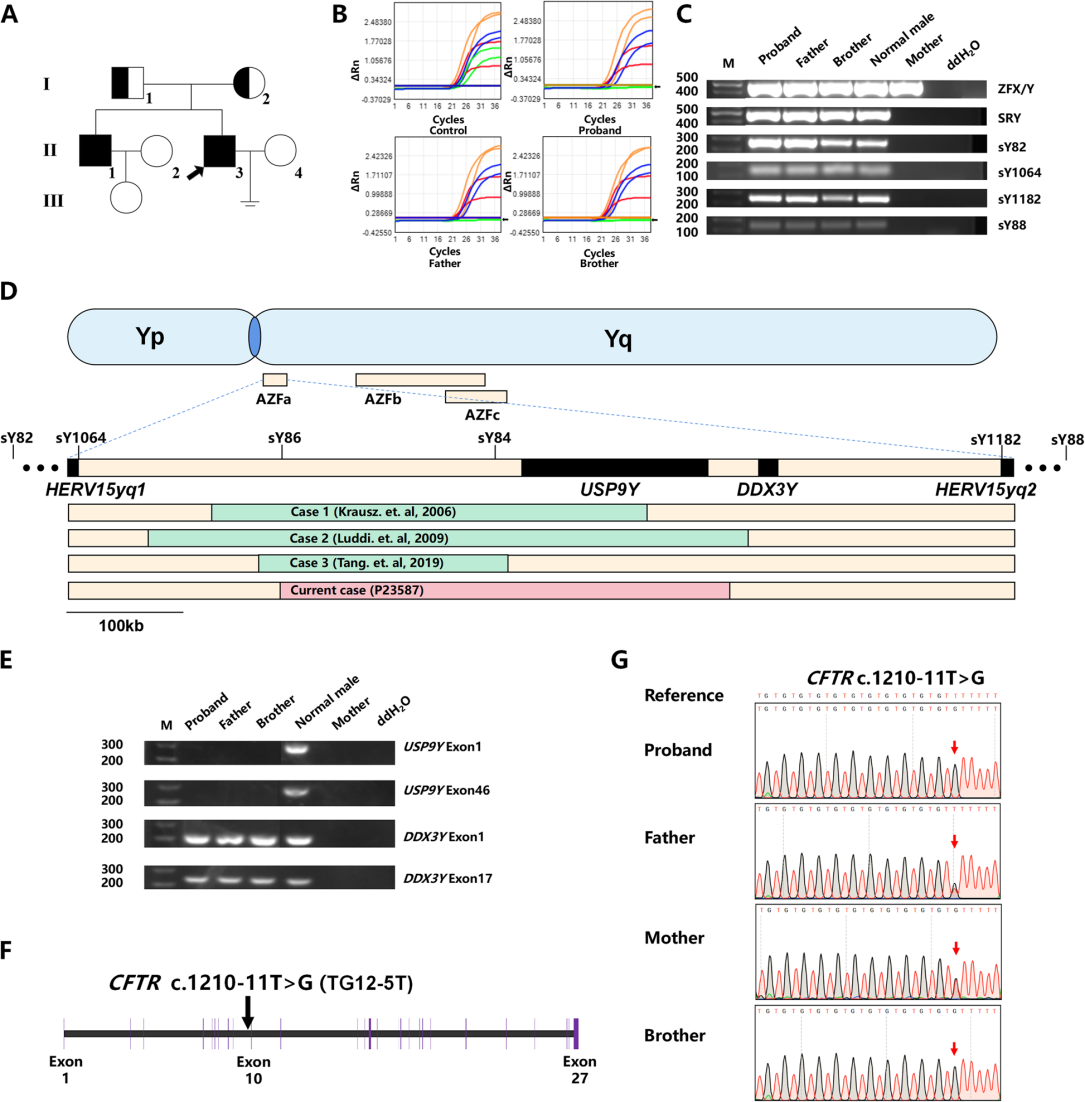
Genetic abnormalities identified by genetic testing.**A**) Pedigree of the family with the *CFTR* variant (c.1210-11 T > G). The arrow indicates the proband. **B**) qPCR amplification plots for various Y-chromosome markers (SRY, ZFX/ZFY in blue; sY84, sY86 in green; sY127, sY134 in orange; sY254, sY255 in red). The plots show the cycle number versus normalized reporter dye fluorescence (ΔRn = Rn—baseline). The black arrows indicate the absence of sY84 and sY86 signals below the detection thresholds. **C**) PCR analyses of AZFa deletion markers (sY82, sY1064 for the proximal border; sY1182, sY88 for the distal border) performed on genomic DNA extracted from peripheral blood samples of the proband, the proband’s father, the proband’s brother, a normal male as the positive control, and the proband’s mother as a negative control. Blank control: ddH_2_O. Molecular-weight standard (M) for comparison. **D**) Schematic diagram of the AZFa, b, c region on the long arm of the human Y chromosome, depicting the partial AZFa deletion in the current case (red box) and previous cases (green box). **E**) PCR analyses of *USP9Y* exon 1, *USP9Y* exon 46, *DDX3Y* exon 1, and *DDX3Y* exon 17 were performed on genomic DNA extracted from peripheral blood samples of the proband, the proband’s father, the proband’s brother, a normal male as the positive control, and the proband’s mother as a negative control. Blank control: ddH_2_O. Molecular-weight standard (M) for comparison. **F**) Depiction of the *CFTR* variant (c.1210-11 T > G) localization in the genome structures. **G**) Sanger sequencing results showing the *CFTR* variant in the pedigree, with variant positions indicated by red arrows

A 32-year-old male (P23587) presented to the Urologic Medical Center of Shanghai General Hospital due to one year of unsuccessful attempts at conception. His clinical course was documented in a timeline (Fig. S1), with relevant data summarized in Table S1. The patient was naturally conceived and had no family history of infertility; notably, his elder brother exhibited normal fertility (Fig. 1A). The proband reported a 10-year smoking history and denied any additional risk factors for infertility, including varicoceles, radiation exposure, chemotherapy, orchitis, cryptorchidism, or testicular cancer. Physical examination revealed a height of 169 cm, weight of 74 kg, and bilateral testicular volumes of 12 mL, with palpably plump bilateral epididymides. Scrotal ultrasound demonstrated bilateral thin netlike ectasia of the corpus and cauda epididymis, alongside the complete absence of the vas deferens. Moreover, transrectal sonography confirmed the absence of bilateral seminal vesicles. In contrast, ultrasonography of the urinary system revealed normal bilateral kidney structures in the proband. Semen analysis revealed a low semen volume of 1.4 mL, a pH of 6.0, and complete azoospermia according to WHO guidelines (6th edition) [19]. Consistent with obstructive pathology, markedly decreased levels of neutral α-glucosidase and fructose were detected in the proband's seminal plasma. All measured serum hormone levels were within the normal range, including follicle-stimulating hormone (FSH) at 2.24 mIU/ml, luteinizing hormone (LH) at 2.44 mIU/ml, testosterone at 4.00 μg/L, and inhibin B (INHB) at 112.74 pg/ml (Table S1). Collectively, the proband was diagnosed with CBAVD and OA. However, the proband's brother and father have not undergone reproductive-specific examinations due to their asymptomatic status and normal reproductive histories; therefore, unilateral vas deferens agenesis cannot be definitively excluded. Meanwhile, routine health check-ups for the brother and father revealed no reported renal abnormalities.

### Genetic testing revealed a partial AZFa deletion and a *CFTR* variant

Genetic tests were performed on the pedigree to investigate the cause of azoospermia. The proband's karyotype was confirmed as 46, XY. Notably, a multiplex qPCR assay detected sY84 and sY86 deletions in the AZFa region of the Y chromosome in the peripheral blood of the proband, his father, and his brother (Fig. 1B). Subsequent deletion extension PCR analyses confirmed a partial AZFa deletion rather than a complete deletion (Fig. 1C). Furthermore, targeted next-generation sequencing (NGS) panel analysis revealed a 384.9 kb deletion in the AZFa region encompassing the *USP9Y* gene without affecting downstream *DDX3Y***(**Table S2). To our knowledge, this is the second longest partial AZFa deletion reported to date [10–12] (Fig. 1D). PCR analyses confirmed the complete deletion of the *USP9Y* gene and the presence of the *DDX3Y* gene in the proband **(**Fig. 1E**)**. Notably, the same deletion was observed in the proband's father and brother, which was consistent with Y-linked inheritance (Fig. 1E). Whole-exome sequencing (WES) revealed a homozygous TG12-5 T variant (c.1210-11 T > G) in intron 9 of *CFTR* that is proximal to the canonical acceptor splice site of exon 10 (Fig. 1F). Sanger sequencing confirmed that the homozygous variant was inherited from the heterozygous parental carriers and that the homozygous *CFTR* variant (c.1210-11 T > G) was also detected in the elder brother (Fig. 1G). Furthermore, RNAfold predicted the formation of more stable RNA hairpins at the intron 9–exon 10 junctions of the *CFTR* variant, probably impeding the interaction with small nuclear ribonucleoproteins (snRNPs) and spliceosomal complex assembly (Fig. S2).

### Histological analysis showed intact spermatogenesis

**Fig. 2:**
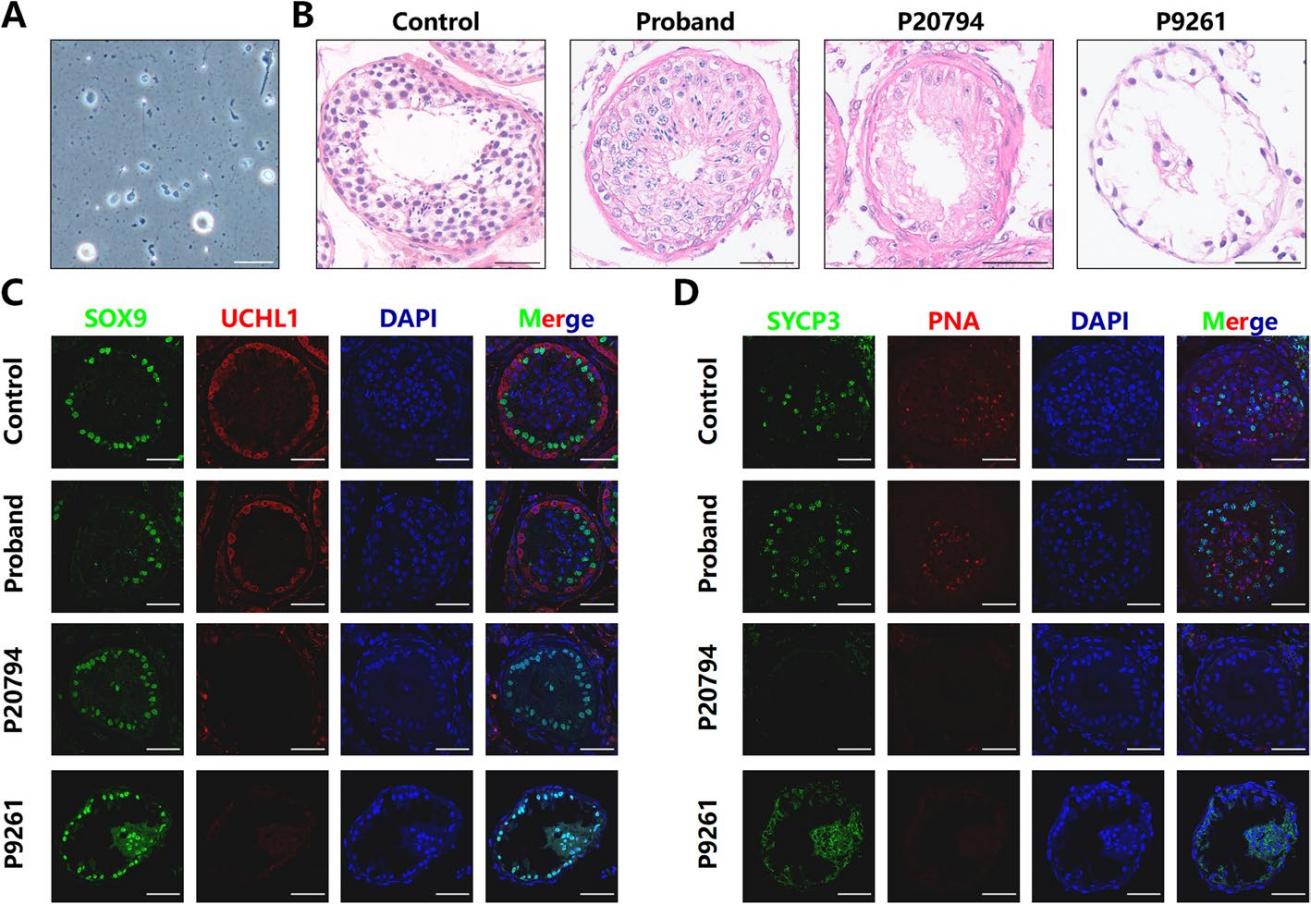
Histological analysis verified intact spermatogenesis in the testis of the proband.** A**) Spermatozoa and germ cells were visibly present in the testicular tissue obtained from the proband during surgery. **B**) Hematoxylin and eosin (HE) staining of testicular tissues from a control OA patient without AZFa deletion (Control), the proband, and two patients with complete AZFa deletion (P9261, P20794). **C**) Immunofluorescence (IF) staining of SOX9 (green) and UCHL1 (red) in the testes of a control OA patient without AZFa deletion (Control), the proband, and two patients with complete AZFa deletion (P9261, P20794).** D**) IF staining of SYCP3 (green) and PNA (red) in the testes of a control OA patient without AZFa deletion (Control), the proband, and two patients with complete AZFa deletion (P9261, P20794). DAPI (blue) was used to stain the cell nuclei in **C, D**. Scale bars = 50 µm

To retrieve sperm, the proband underwent the microdissection testicular sperm extraction (micro-TESE) procedure after thorough preoperative examinations. Intraoperatively, the bilateral absence of the vas deferens and normal seminiferous tubules were confirmed. The bilaterally obtained testicular tissues were minced mechanically and then examined via phase-contrast microscopy, revealing 3–5 immotile sperm per 20 × magnification field. These sperm were then frozen and preserved (Fig. 2A). Thereafter, the couple proceeded with intracytoplasmic sperm injection (ICSI), achieving fertilization and embryo development. Following embryo transfer, implantation occurred successfully, and the female partner is currently experiencing a normal pregnancy. Histopathological analysis confirmed intact spermatogenesis in the testicular tissue. Hematoxylin and eosin (HE) staining revealed the well-organized arrangement of germ cells within the proband’s seminiferous epithelium, which was comparable to that of a control OA patient without AZFa deletion. However, germ cells were absent in two patients with complete AZFa deletion (P9261, P20794) (Fig. 2B). Immunofluorescence (IF) staining further identified Sertoli cells, marked by SOX9, in the testes of the proband, P9261, P20794, and the control. Additionally, the expression of UCHL1, SYCP3, and PNA, indicating spermatogonial stem cells, spermatocytes, and spermatids, respectively, was positive in the proband's testis, resembling those in the controls. In contrast, these markers were not expressed in the testes of P9261 and P20794 (Fig. 2C, D).

## Methods

The detailed methodologies used for WES, polymerase chain reaction (PCR) amplification, and primer sequences are provided in the Supplementary Methods. In summary, clinical information was collected from the proband. Genomic DNA was extracted from peripheral blood samples of a control individual and members of the proband's pedigree. qPCR assays were subsequently employed to screen for Y chromosome microdeletions, and deletion extension PCR analyses were conducted according to the 2023 EAA/EMQN guideline, employing standard STS markers and specific primers. The AZF region of the Y chromosome was further investigated using a targeted NGS panel. PCR analyses were performed to evaluate the presence of the *USP9Y* and *DDX3Y* genes. Additionally, HE and IF staining were conducted on testicular tissues from a control OA patient without AZFa deletion, the proband, and two patients with complete AZFa deletion (P9261, P20794). The clinical features of the patients are shown in Table S3. The primer sequences used for PCR amplification are detailed in Table S4.

## Discussion

In this study, we identified an approximately 384.9 kb AZFa microdeletion in an azoospermia-affected patient, which specifically affects *USP9Y* without involving *DDX3Y*, alongside a homozygous *CFTR* variant. To the best of our knowledge, this is the first report of a partial AZFa microdeletion combined with a homozygous *CFTR* variant (c.1210-11 T > G). This is also the second case of intact spermatogenesis with complete *USP9Y* deletion [12]. Compared to the previous report, this case offers more comprehensive evidence on the histology of testicular tissue. Crucially, the proband's testicles exhibited intact spermatogenesis on the basis of testicular histological morphology, indicating that *DDX3Y*, rather than *USP9Y*, plays a key role in spermatogenesis within the AZFa region.

*USP9Y*, a member of the deubiquitinating gene family, encodes a protein with ubiquitin C-terminal hydrolase activity. This protein potentially regulates protein turnover by preventing degradation through the removal of ubiquitin from protein-ubiquitin conjugates [20]. In humans, the isolated absence of *USP9Y* has been linked to a spectrum of phenotypes, ranging from azoospermia with hypospermatogenesis to severe oligozoospermia and normozoospermia [12, 21, 22]. However, *USP9Y* is expressed primarily in spermatids, suggesting that it might act as a fine-tuner of germ cell maturation rather than an essential player in complete spermatogenesis [12, 13]. This is supported by the fertility of chimpanzees and bonobos with inactive *USP9Y* [23, 24]. In the absence of *USP9Y*, its function might be compensated by its X-homolog, *USP9X*, which shares 91% identity, or potentially by other deubiquitinating enzymes [25, 26].

The other gene, *DDX3Y,* which is predominantly expressed in spermatogonia, is assumed to be crucial for spermatogenesis within the AZFa region [27]. Loss-of-function variants in *DDX3Y* abolish the expression of the C-terminal helicase domain, leading to transcript degradation and the SCO phenotype observed in the men [28]. Functional studies have shown that the introduction of *DDX3Y* can restore germ cell formation in individuals with AZFa deletions, reinforcing its essentiality in spermatogenesis [29].

In this case, the homozygous TG12-5 T *CFTR* variant in the CBAVD-affected proband and his fertile brother indicated pathogenicity with incomplete penetrance. Unlike many genetic disorders, *CFTR* variants do not conform to an “all or none” disease paradigm. Therefore, phenotypic severity can vary even among individuals sharing identical *CFTR* genotypes and is influenced by factors such as CFTR residual activity, endocrine regulation, epigenetics, or the environment [16, 30, 31]. The 5 T variant reduces intron 9 splicing efficiency, leading to ~ 50% full-length CFTR vs. ~ 75% in 7 T/9 T carriers [18]. Specifically, the longer TG tract (12–13 repeats) with 5 T decreases CFTR production to ~ 25% [18]. TG12-5 T heterozygous variants are common in CBAVD-affected patients [32–38], but homozygous variants are relatively rare [39]. Interestingly, these homozygotes can have normal vas deferens [17]. In a *CFTR* gene sequencing cohort, TG12-5 T homozygotes were absent in CF/CFTR-RD suspects but present in 108 low-suspicion individuals [18]. The homozygous TG12-5 T variant was predicted to alter the local secondary structure at the intron 9-exon 10 junction. This structural alteration is expected to disrupt the spliceosomal complex assembly and facilitate mRNA degradation via the no-go degradation (NGD) pathway, reducing splicing efficiency in exon 10 and functional CFTR protein expression [40–42].

## Conclusion

This study clarifies the phenotypic impact of *USP9Y* and *DDX3Y* in the AZFa region, highlighting the essential role of *DDX3Y* in spermatogenesis. The homozygous TG12-5 T *CFTR* variant demonstrates the intricacies of genotype‒phenotype correlations in the context of CBAVD. Comprehensive genetic screening and targeted *DDX3Y* analysis are crucial for AZFa microdeletion diagnosis and management. Future research should delve deeper into the mechanism of *DDX3Y* in spermatogenesis.

## Supplementary Information

Supplementary Material 1

## Data Availability

No datasets were generated or analysed during the current study.

## Abbreviations

NOA: Non-obstructive azoospermia
OA: Obstructive azoospermia
AZF: Azoospermia factor
NAHR: Nonallelic homologous recombination
SCO: Sertoli cell-only
STS: Sequence-tagged sites
CBAVD: Congenital bilateral absence of the vas deferens
CFTR-RDs: CFTR-related disorders
FSH: Follicle-stimulating hormone
LH: Luteinizing hormone
INHB: Inhibin B
HE: Hematoxylin and eosin
IF: Immunofluorescence
PCR: Polymerase chain reaction
WES: Whole-exome sequencing
NGS: Next-generation sequencing
NGD: No-go degradation
