# Supplementary material for: Intact spermatogenesis in an azoospermic patient with AZFa (sY84 and sY86) microdeletion and a homozygous TG12-5T variant in CFTR
Source: Basic Clin Androl. 2025 Apr 1;35:13. doi: 10.1186/s12610-025-00260-7 (PMC11963436; doi:10.1186/s12610-025-00260-7)
Supplement: Supplementary file 1 — Supplementary Material 1 [file 12610_2025_260_MOESM1_ESM.docx]

**SUPPLEMENTARY MATERIAL**

**SUPPLEMENTARY METHODS**

**Whole-exome sequencing**

Genomic DNA was extracted from the blood of the proband with NOA via the TIANamp Blood DNA Kit (TIANGEN Biotech, Beijing, China) according to the manufacturer’s instructions. DNA was fragmented through Covaris-focused ultrasonication. Known exons and exon‒intron boundary sequences were captured via the xGen Exome Research Panel (Integrated DNA Technologies, Coralville, IA, USA), and the resulting DNA libraries were sequenced following the manufacturer’s instructions. Sequencing was performed on a HiSeq×10 platform (Illumina, San Diego, CA, USA). The sequencing reads were aligned to the human genome (GRCh37/hg19) via the Burrows–Wheeler Aligner (BWA). Both SNVs and indels within the captured coding exonic intervals were called via GATK, Platype, VarScan, LoFreq, FreeBayes, SNVer, SAMtools, and VarDict. The variants were filtered and annotated via ANNOVAR software.

Genetic variants with allele frequencies higher than 1% according to the GnomAD and 1000 Genomes Project were excluded, and the intronic, upstream, and downstream variants were removed. Nonsense, frameshift, essential splice site, and potentially deleterious missense (SIFT, PolyPhen-2, and MutationTaster) variants were retained for further analysis. A gene with an allele with potentially deleterious missense mutations or LoF mutations was retained because it was assumed to be MA. We also compared the candidate genes with known pathogenic genes for azoospermia in mice (http://www.informatics.jax.org/mgihome/homepages/) and testis-enriched genes in the database (http://www.proteinatlas.org/). The aforementioned sequencing and bioinformatic analyses were conducted together with the Nuprobe Company (Shanghai, China). The datasets used and analyzed during the current study are available from the corresponding author upon reasonable request.

**PCR amplification**

The primers used are shown in **Table S4**. The PCR products were bidirectionally sequenced via Sanger sequencing via a 3730xl DNA Analyzer (Applied Biosystems, Foster City, CA, USA).

**Immunofluorescence (IF)**

Testicular biopsies were obtained from the proband with NOA. Testicular tissue was fixed overnight in 4% paraformaldehyde at 4°C and then embedded in warm paraffin (60°C). The tissue blocks were cut into 5-μm-thick sections and mounted onto slides. The tissue sections were dewaxed in xylene, rehydrated in a descending alcohol gradient, and heated in sodium citrate buffer (90°C–98°C) for 15 min for antigen retrieval. After being blocked with 5% normal donkey serum for 1 hour at room temperature, the sections were incubated overnight with anti-SOX9 (dilution: 1:500, catalog number: AB5535; Millipore), anti-UCHL1 (dilution: 1:200, catalog number: MCA4750; Bio-Rad), anti-SYCP3 (dilution: 1:25, catalog number: AF3750; R&D) and anti-PNA (dilution: 1:500, catalog number: L-32459; Life) secondary antibodies at 4°C. The sections were washed three times with PBS-Tween and incubated with highly cross-adsorbed secondary antibodies conjugated with Alexa Fluor 488 or Alexa Fluor 594 (dilution: 1:400, Life Technologies/Thermo Fisher Scientific) for 1 hour at room temperature. After the final three washes, the sections were counterstained with 4’,6-diamidino-2-phenylindole (DAPI) to label the nuclei, and images were captured with a fluorescence microscope (Leica, Wetzlarler, Germany).

**Patient Perspective**

Patient P23587, a 32-year-old male, shared his experience and feelings. He described, "I have always wanted to be a father, but in the past year, despite our efforts as a couple, we have never been able to achieve it. When I faced the reality of azoospermia, I felt a profound sense of shock and uncertainty. The journey from diagnosis to understanding the genetic complexities of my condition has been emotionally taxing. The support from my family and the medical team has been invaluable, and their empathy has made the process more bearable. I am hopeful about the future and the advancements in treatment that could improve my situation. To others in a similar position, I would say that it is important to stay informed and seek support from those around you."

**FIGURE LEGENDS**

**Fig. S1 Timeline illustrating the clinical course of the patient**

Graphic time course of events for the case. Major events are indicated in black.

Abbreviations: OA (obstructive azoospermia); CBAVD (congenital bilateral absence of the vas deferens); AZF (azoospermia factor); PCR (polymerase chain reaction); WES (whole-exome sequencing); NGS (next-generation sequencing); micro-TESE (microdissection testicular sperm extraction); HE (hematoxylin and eosin); IF (immunofluorescence).

**Fig. S2 Predicted RNA secondary structure changes at the *CFTR* intron 9-exon 10 junction via RNAfold**

The diagram illustrates the predicted RNA secondary structure changes at the intron 9--exon 10 junctions of the *CFTR* gene for both the reference sequence (“*CFTR* (Reference)”) and the proband carrying the homozygous TG12--5T variant (“*CFTR* (Proband)”). Nucleotide bases are color-coded (adenine [A] in green, cytosine [C] in yellow, guanine [G] in red, and uracil [U] in blue). The splicing direction is indicated by arrows. Key nucleotide positions (c.1210, c.1216, c.1226, c.1236, c.1210-14, c.1210-11, c.1210-4) are labeled, highlighting structural differences due to the variant. RNAfold analysis suggested the formation of more stable RNA hairpins in the proband, potentially affecting splicing efficiency. Abbreviations: *CFTR* (cystic fibrosis transmembrane conductance regulator).

**TABLE S1 Clinical features of the proband**

**TABLE S2 Partial AZFa Deletion Identified in the Proband Using Targeted Next-Generation Sequencing Panel Analysis**

**TABLE S3 Clinical features of three patients with AZFa sY84 and sY86 deletions**

**TABLE S4 Primer design for PCR amplification**
